# Getting Health Hazards of Inhaled Nano/Microplastics into Focus: Expectations and Challenges

**DOI:** 10.1021/acs.est.3c00029

**Published:** 2023-02-22

**Authors:** Guodong Cao, Zongwei Cai

**Affiliations:** State Key Laboratory of Environmental and Biological Analysis, Department of Chemistry, Hong Kong Baptist University, Hong Kong SAR 999077, China

**Keywords:** inhaled nano/microplastics, human health, 2D cell culture, 3D spheroids, human organoids

Microplastics (<5 mm in diameter, including <1 μm nanosized plastics) have been ubiquitously detected in marine, freshwater, terrestrial, and atmospheric systems since 2004.^1^ Thereafter, understanding the biological consequence of these plastic particles has become of interest to many areas of science. A growing body of evidence indicates that microplastics are detrimental to a wide range of living organisms, including plankton, fish, microorganisms, plants, and rodents. Newer data suggest that microplastics are moving from the environment to the human body, as demonstrated by their presence in human lungs, placenta, and stool, which has raised significant concerns about their hazards and human health implications. Ingestion and inhalation are considered the major routes of human exposure to microplastics. Although there have been more toxicological studies regarding the biotoxicity of microplastics in recent years, there is a paucity of research for interrogating the toxicological effects of airborne microplastics on humans via inhalation.

According to a survey conducted by Cox et al., humans may consume 74000–121000 microplastic particles per year, where inhalation contributes approximately half of the annual exposure estimates.^2^ In line with this finding, researchers in the United Kingdom compared human consumption of microplastics via ingestion of contaminated mussels to that of household fiber fallout during a meal.^3^ Their results indicated the latter poses a greater risk. Additionally, there is compelling evidence that workers processing nylon, polyester, and polyamide fibers in the United States, Canada, and The Netherlands exhibited a higher prevalence of respiratory irritation, with severe symptoms being coughing, dyspnea, occupational asthma, and interstitial lung disease, implying the health hazards of high-dose exposure of inhaled microplastics.^4^ Our recent study also demonstrated the role of the size of nanoscale plastics in the internalization in and mitochondrial damage to human respiratory cells.^5^ However, much information is still lacking about the toxicological effects and risks of low-level exposure of inhaled microplastics as occurs in the general population under realistic exposure scenarios. Unlike other air quality indices, such as PM_2.5_ and PM_10_, the widespread use and continuous abrasion of plastic-related products seems to have eliminated the air pollution inequality of airborne microplastics between developing and developed countries, where high levels of microplastics in the atmosphere have been found in London (England), Paris (France), Tehran (Iran), Shanghai (China), and Hamburg (Germany),^6,7^ which poses a threat to global health and imposes a significant regulatory burden. In view of these facts, we thus call for extensive research to interrogate the health hazards and molecular determinants of inhaled microplastics by using either canonical or new toxicological models.

**Figure 1:**
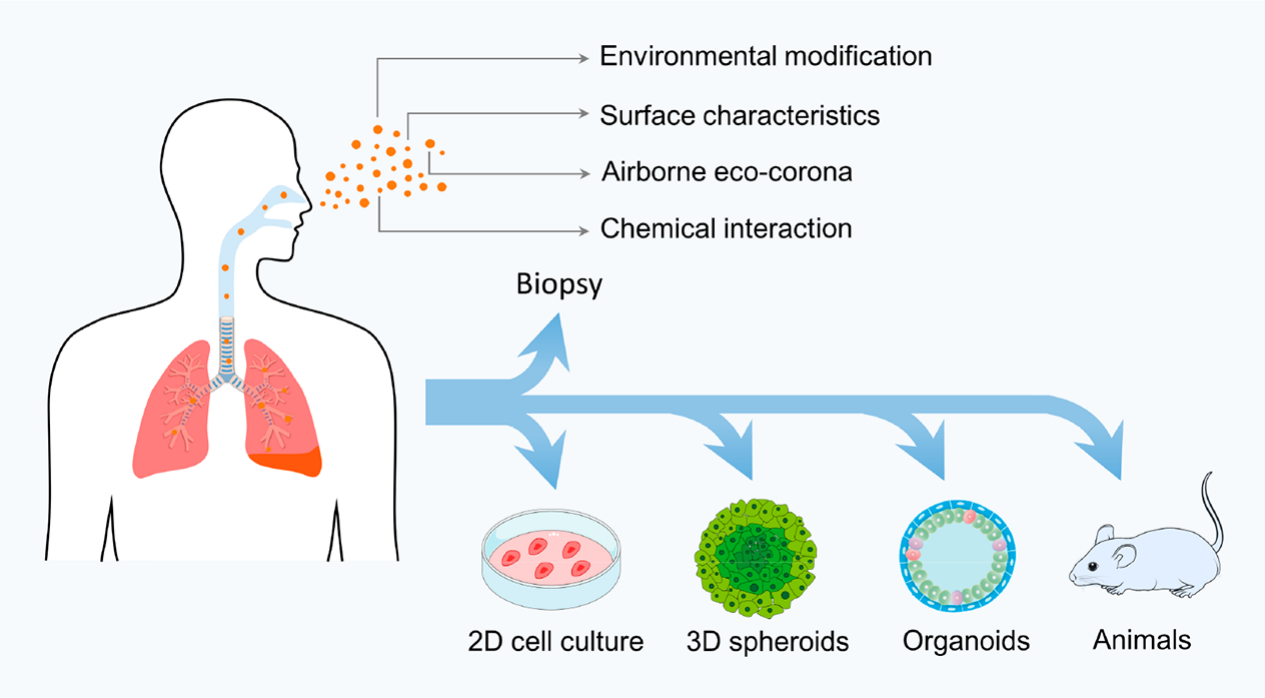
Various *in vitro* and *in vivo* models along with the consideration of environmental complexity for addressing the uncertainty surrounding the risk of inhaled nano/microplastics.

There are some obstacles and challenges to overcome in seeking answers about inhaled microplastic-induced toxicity. Available data suggest that various physicochemical properties of microplastics, such as polymer types, sizes, surface areas, and functional groups, have an inevitable impact on their biotoxicity. The situation becomes more complicated when plastic particles enter the atmospheric environment, which may undergo a variety of degradation and erosion processes, such as hydrolysis, photodegradation, and mechanical disintegration, resulting in large variability in their morphology and surface characteristics (Figure 1). Parallel evidence has indicated that the abundance and types of airborne microplastics might vary over urban, suburban, and remote areas.^7^ Consequently, these confounding factors lead to a striking discrepancy between microplastic particles found in nature and made in the laboratory; the latter have been predominantly used in toxicological studies. Another dimension of toxicological concern is that atmospheric microplastics may act as vectors to transfer toxicants in the air, such as heavy metals and organic chemicals. These toxic hazards, along with the artificial and natural additives of plastic itself (e.g., stabilizers, plasticizers, flame retardants, and antimicrobial agents), may lead to complex chemical interaction in a variety of ways, which may magnify or mitigate the biological effects of inhaled microplastics. Recent studies have found that microplastics in the aquatic environment tend to form eco-corona upon attachment of microorganisms and biomolecules to their surfaces, which has been shown to enhance the internalization of microplastics into cells and organisms.^8^ However, it is still unclear whether such modification will occur in airborne microplastics. As a corollary, delicately designed experiments are needed to address the chemical complexity of microplastics under more realistic exposure scenarios. We thereby recommend using naturally aged microplastics in the atmosphere to conduct *in vitro* and *in vivo* toxicological studies. The rational use of computational tools (e.g., machine learning and artificial intelligence) combined with multifactorial experimental designs would also provide vivid information for evaluating the contribution of relevant confounding factors of inhaled microplastics (e.g., dose, size, shape, and chemicals interact) to the effects on human health.

More promising but still problematic is assessment of health impacts of inhaled microplastics in the human respiratory system. A straightforward approach is to measure the exposure levels of microplastics deposited on lung tissues or exudated into pleural effusion. However, the current analytical methods still have limitations for characterizing small plastics (<3 μm) in such biological samples. As a consequence, *in vitro* and *in vivo* models have become increasingly important for interrogating the possible toxicity of inhaled microplastics (Figure 1). Two-dimensional (2D) and three-dimensional (3D) respiratory cell cultures provide a feasible means of investigating the internalization, permeation, and translocation of inhaled microplastics at the cellular level. The generation and synergistic use of human lung organoids that physiologically and functionally recapitulate the organizational structures of alveolars, airways, and lung buds can yield more vivid information implying the localized and specific responses to inhaled microplastic stimuli. In addition, *in vivo* inhalation studies that simulate human exposure scenarios of inhaled microplastics are also important to reflect the complex interplay of different types of cells and/or different organ systems. Various exposure models, such as instillation, a short-term inhalation study (STIS), and a real-ambient exposure system, can be used alone or integrated with computational tools to better understand the metabolic signatures associated with inhaled microplastic exposure.

At this point, it is worth emphasizing that regulatory guidance about hazard assessment of inhaled microplastics has not yet been finalized. This absence calls for expedited studies to address these open questions, knowledge gaps, and uncertainties regarding the toxicity of inhaled microplastics. However, in a real-world setting, due to the complexity of airborne microplastics, it is imperative to utilize integrated testing strategies to elucidate the toxic mechanisms of inhaled microplastics. There are still needs for highly standardized and streamlined workflows with an integrated analytical platform to gather multiangle information about airborne microplastics, such as the shape, size, surface area, mass concentration, and number concentration, as a prerequisite for elaborating their exposure dose–response relationships in both *in vitro* and *in vivo* models. In addition, studies with cells in culture and in rodents should be performed under more realistic exposure scenarios to extrapolate their toxicological effects to humans. Despite the challenges ahead, now is an opportune time to identify near-term and long-term risks of inhaled microplastics and establish the principles for mitigating their environmental impacts in the near future.
